# Synthesis of Good Electrical Conductivity of CoCe-BTC/PEDOT for Ultrahigh Selectivity of NO_2_ Detection

**DOI:** 10.3390/s22186891

**Published:** 2022-09-13

**Authors:** Xiaoting Zha, Runhui Xi, Yuanyuan Wu, Jianhua Xu, Yajie Yang

**Affiliations:** 1 School of Optoelectronic Science and Engineering, University of Electronic Science and Technology of China, Chengdu 610054, China; 2 Yangtze Delta Region Institute (Huzhou), University of Electronic Science and Technology of China, Huzhou 313001, China

**Keywords:** CoCe-BTC, PEDOT, gas sensor, NO_2_

## Abstract

Metal–organic frameworks (MOFs) have broad application prospects in the development of efficient, sensitive and single select gas sensors. However, in order to construct a chemical resistance gas sensor based on MOFs, the problem of poor conductivity of MOFs must be solved. In this work, we synthesized CoCe-BTC, which based on the organic ligands of trimesic acid (H_3_BTC) by the water bath method and prepared CoCe-BTC/PEDOT composite film on an interdigital electrode by the spin coating process. Compared with pure MOF material, the conductivity of CoCe-BTC/PEDOT is significantly improved. Under a dry room temperature environment and N_2_ as the carrier gas, the response of the sensor to NO_2_ is about 1.2 times that of pure PEDOT and has a shorter response time. It has great repeatability and selectivity and shows a dynamic response with the change of NO_2_ gas concentration (5–50 ppm).

## 1. Introduction

After “coal smoke pollution” and “photochemical smoke pollution”, there is the third environmental pollution dominated by “indoor air pollution”. The concepts of environmental protection and sustainable development are increasingly recognized. Therefore, researchers have put forward higher requirements for sensor monitoring of air pollution. As the core of the sensor, gas sensitive materials with high sensitivity and high selectivity are the focus of gas sensor research and development.

Metal–organic frameworks (MOFs) are reticular framework materials formed by coordination between metal ions and organic ligands. MOFs have the advantages of diverse structures, high porosity, large specific surface area and many active sites. The adjustability of metal ions and organic connectors gives MOFs designability in topology, porosity (pore size and geometry) and specific responsiveness to external stimuli.

In recent years, research of chemical resistance sensors based on MOFs has developed rapidly [1,2]. These sensors act through resistance changes caused by the reaction or adsorption between the target gas and the surface of MOFs. Although MOFs as gas sensing materials have good porous characteristics and surface activity, the poor conductivity of MOFs greatly inhibits the improvement of their sensitive characteristics, and it is difficult to achieve a rapid response to external stimuli, which limits their practical applications in sensitive elements, especially in sensitive elements based on resistance change mechanisms. Therefore, to realize the development of MOFs in the field of electronics and sensitive materials, solving the problem of low conductivity is the key.

When improving the conductivity of MOFs by combining them with conductive polymers, a donor–acceptor structure can be formed between MOFs and conductive polymers. More importantly, the interface enhancement effect and synergistic effect between MOFs and conductive polymers make the composite reflect a series of characteristics better than pure MOFs, such as enhanced electromagnetic absorption [3], enhanced biological sensitivity [4,5], enhanced gas sensitivity [6,7,8], etc.

Polymetallic MOFs have all the advantages and applications of single metal MOFs. Considering the importance of metal sites of MOFs in playing various roles, a new strategy to improve the performance of MOFs by doping other metals in the skeleton has been proposed in recent years. Dai et al. reported the synthesis of bimetallic NiCu-BTC [9], ZnCu-BTC [10], VCu-BTC [11] and trimetallic NiZnCu-BTC [12] (BTC represents MOFs with trimesic acid H_3_BTC as the organic ligand) by the solvothermal method, which confirmed this view by comparison [13].

Poly3,4-ethylenedioxythiophene (PEDOT) is formed by polymerization of 3,4-ethylenedioxythiophene (EDOT) monomer. It has good electrochemical reversibility, significant conductivity in the doped state, low oxidation potential, narrow band gap and excellent chemical, optical, thermal and mechanical stability. It is widely used in many fields, such as supercapacitors, batteries, display equipment and sensors.

In this paper, the CoCe-BTC/PEDOT composite sensor is simply and efficiently prepared by the spin-coating method. The sensor realizes the significant response of NO_2_ gas at room temperature and has good repeatability and selectivity (compared with the common interfering gases SO_2_, CO_2_, NO and NH_3_). This work provides a good experimental basis and theoretical reference for the preparation and sensitivity enhancement of low-resistance MOF composites.

## 2. Experimental

### 2.1. Synthesis of CoCe-BTC

**Figure 1:**
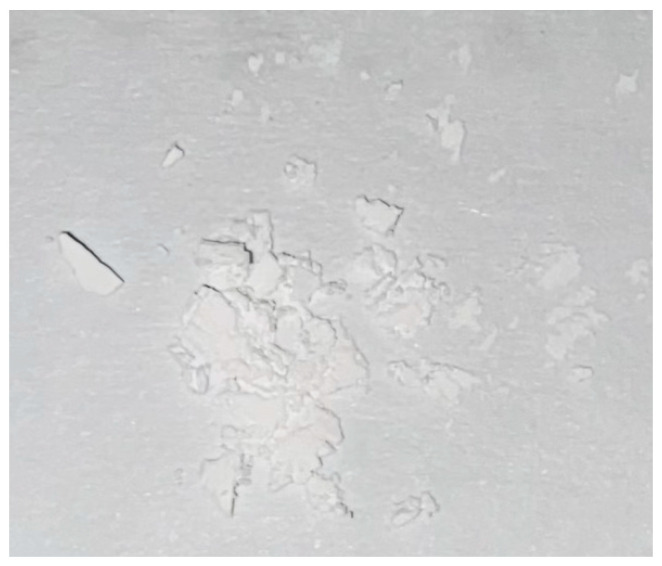
The photograph of CoCe-BTC powder.

Co(NO_3_)_2_·6H_2_O (72 mg) and Ce(NO_3_)_3_·6H_2_O (975 mg) were dissolved in 5 mL H_2_O. Then 1,3,5-benzenetricarboxylic acid (525 mg) was dissolved in the mixed solution of ethanol (100 mL) and H_2_O (100 mL). The above solutions were mixed and heated in a 90 °C water bath with constant stirring. Then the solution was centrifuged and washed with ethanol 3 times. Figure 1 shows white powder obtained after vacuum drying.

### 2.2. Fabrication of Gas Sensor

**Figure 2:**
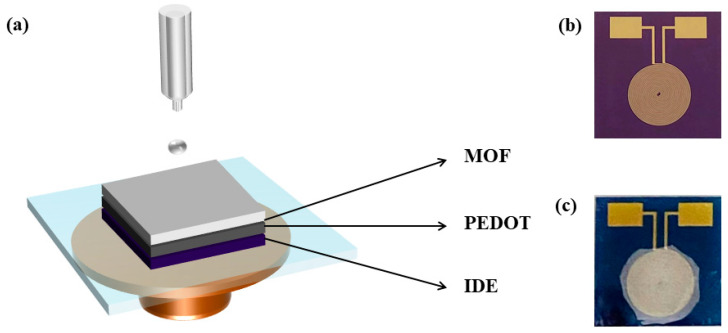
Preparation of composite films: (**a**) the scheme of the spin coating method; (**b**) photograph of IDE; (**c**) IDE coated in CoCe-BTC/PEDOT.

CoCe-BTC/PEDOT composite films were prepared with an interdigital electrode (IDE) by the spin-coating method (Figure 2a), as shown in Figure 2b. Solution I: 30 mg CoCe-BTC was dissolved in 1 mL ethanol. Solution II: PEDOT aqueous dispersion was dried and redispersed in 1 mL ethanol. Then 10 μL solution I was taken and dropped onto the interdigital electrode and spin coated for 5 layers and dried. The above steps were repeated again. Then, 10 μL solution II was dropped onto the interdigital electrode. Figure 2c shows the device obtained after drying.

### 2.3. Material Characterizations

An X-ray diffractometer (XRD) was used to analyze the samples by X-ray diffraction and observe their crystallization. The morphology of CoCe BTC powder and CoCe BTC/PEDOT composite film was observed using a Gemini SEM 300. The pore size distribution and N_2_ adsorption/desorption isotherms of CoCe-BTC powder were obtained by ASAP 2020. An Ultim Max 40 was used to observe the element distribution on the surface of the composite film.

### 2.4. Gas Sensitivity Test Systems

**Figure 3:**
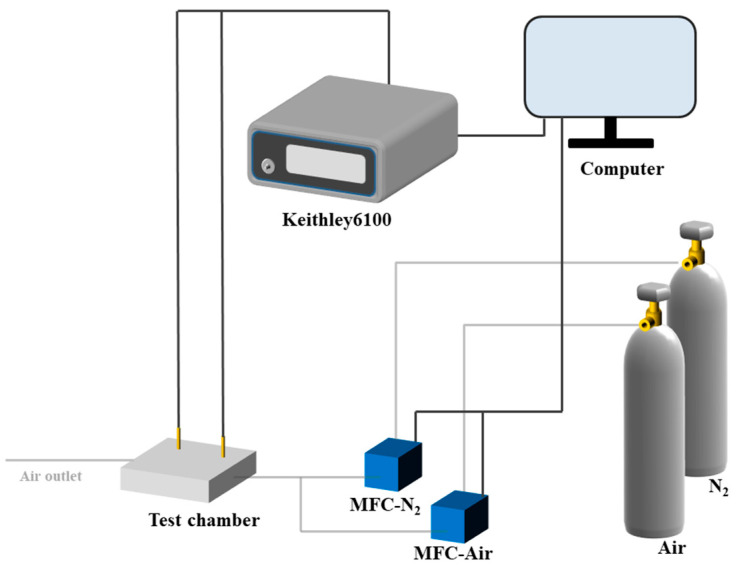
Gas sensitivity test system.

The test system is shown in Figure 3. An AITOLY MFC 300 gas flowmeter was configured to deliver target gas with a specific concentration into the test chamber. A Keithley 6100 was used to collect the dynamic resistance data of sensors in different gas atmospheres. The response (S) of the sensor was defined as S = (Ra − Rg)/Ra. where Ra and Rg represent the resistance in N_2_ and the gas, respectively. The response and recovery times were defined as the time taken to reach 90% of the total resistance change in the case of response and recovery processes, respectively. 

## 3. Results and Discussion

### 3.1. Structure Characterization

**Figure 4:**
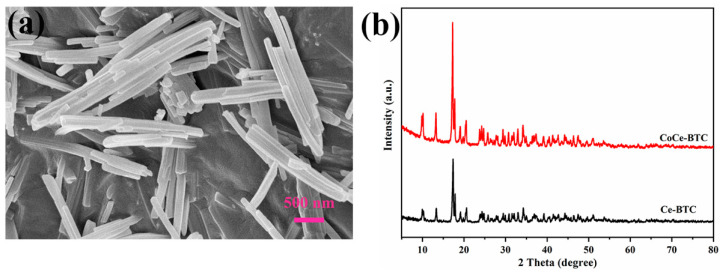
(**a**) The SEM image of CoCe-BTC. (**b**) Powder XRD patterns of CoCe-BTC and Ce-BTC.

**Figure 5:**
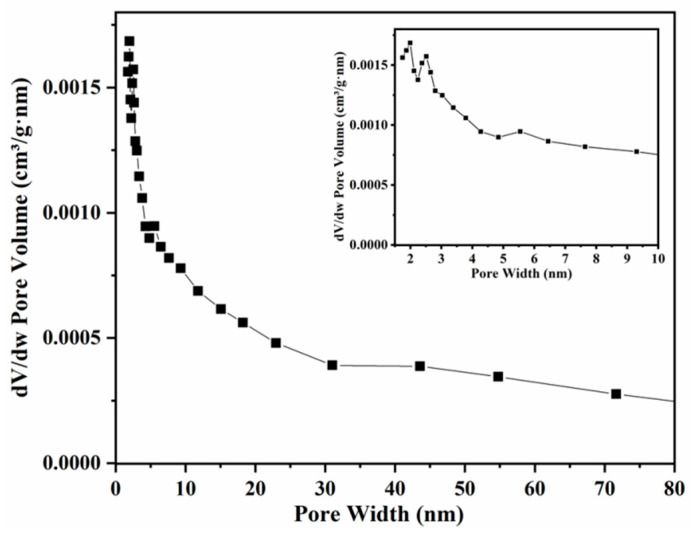
BJH pore size distribution curve of CoCe-BTC.

**Figure 6:**
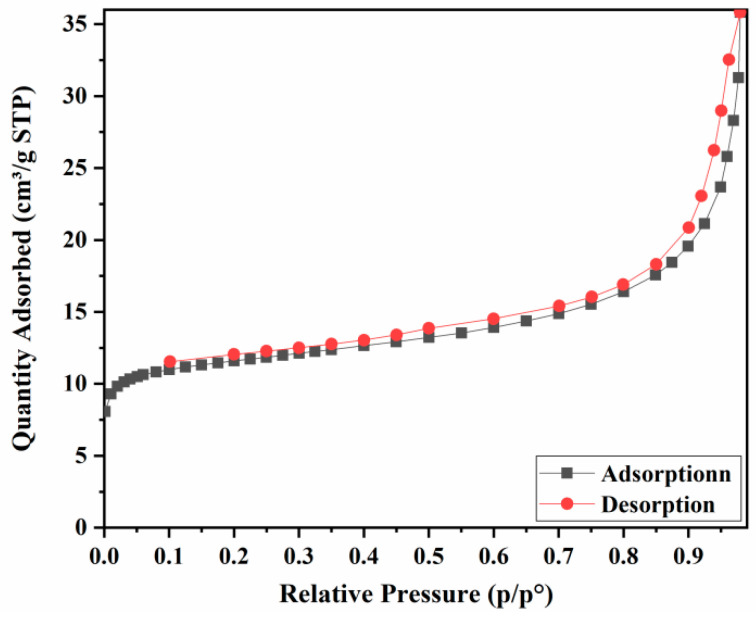
N_2_ adsorption/desorption isotherms of CoCe-BTC.

A scanning electron micrograph showed that the CoCe-BTC powders were presented as columnar strips (Figure 4a). The diameter of columnar CoCe-BTC was about 160 nm, showing its excellent nanometer size effect. The powder XRD patterns (Figure 4b) illustrated that the diffraction peak of Ce-BTC was consistent with the results reported in [14] Remarkably, the XRD pattern of Ce-BTC was isostructural to the simulated La-MOF (CCDC 290771). In this structure, Ce was coordinated with three homoparbenzoic acid and six water. Furthermore, the XRD diffraction peak of CoCe-BTC was similar to that of Ce-BTC, indicating that Co-BTC is an amorphous structure, and the presence of Co did not change the crystal structure of CoCe-BTC [15]. Figure 5 shows that the pore sizes of CoCe-BTC were mainly concentrated at 1.99 nm and 2.51 nm. Figure 6 displays a typical type IV isotherm with the type-H_3_ hysteresis loop, which is related to the formation of numerous mesoporous structures. The specific surface area of the CoCe-BTC was 14.56 m^2^ g^−1^. The pore structures were irregular and diverse. 

**Figure 7:**
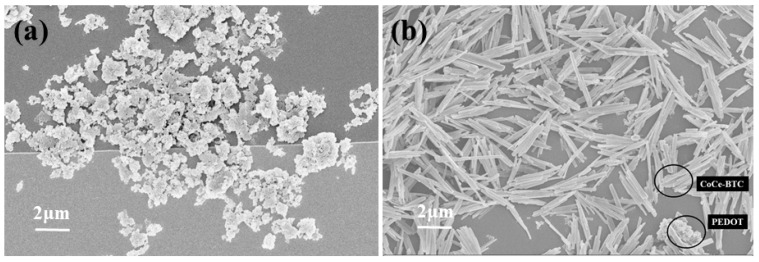
SEM images of as-synthesized samples on IDEs: (**a**) PEDOT; (**b**) CoCe-BTC/PEDOT.

**Figure 8:**
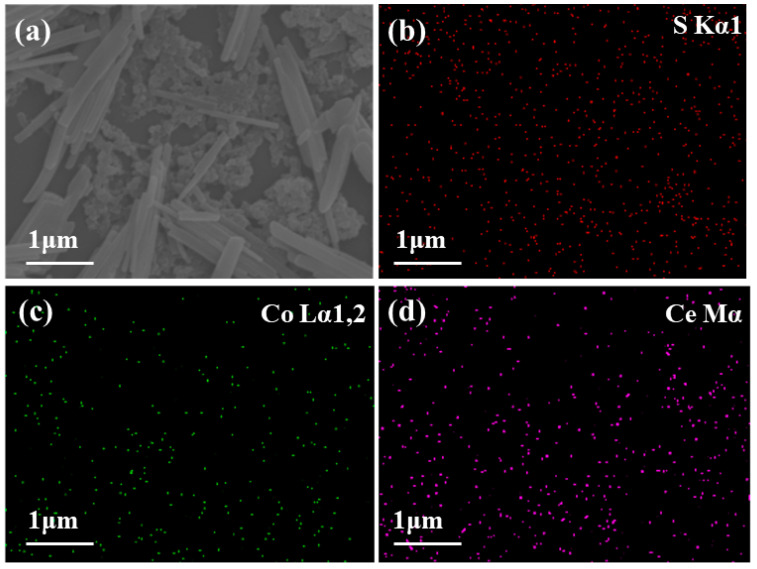
EDS images of as-synthesized samples on IDEs: (**a**) electron image; (**b**) S; (**c**) Co; (**d**) Ce.

It can be seen in Figure 7 that the PEDOT layer on the surface of IDE overlapped with the CoCe-BTC layer, and the interlayer was in great contact, which was conducive to the synergy between the two materials. The unsmooth surface provided more adsorption sites for gas molecules, and the gap between materials was conducive to the flow of gas molecules. The distribution of Ce, Co and S elements on the surface of IDE was dense and uniform (Figure 8), which shows that the composite material was successfully prepared.

### 3.2. Repeatability of Sensors

**Figure 9:**
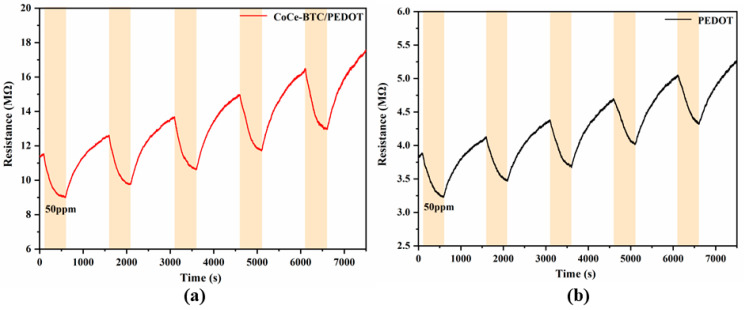
Dynamic response curves of the (**a**) PEDOT and (**b**) CoCe-BTC/PEDOT gas sensors to 50 ppm NO_2_ for 5 cycles at room temperature.

The sensor to was exposed to 50 ppm NO_2_ for 5 cycles in a dry room temperature environment, and the results are shown in Figure 9. When exposed to NO_2_, the sensors showed a decrease in resistance. Both of them had great repeatability. In addition, the response of CoCe-BTC/PEDOT was larger and was about 1.2 times than that of PEDOT. Furthermore, the response time of CoCe-BTC/PEDOT was shorter. 

### 3.3. Dynamic Response to Different Concentrations of NO_2_

**Figure 10:**
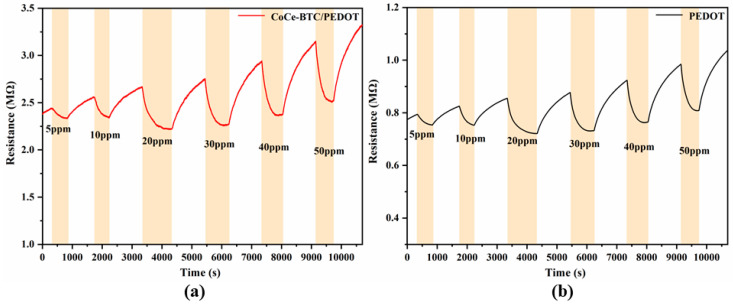
Transient resistance curves of the (**a**) PEDOT and (**b**) CoCe-BTC/PEDOT gas sensors in response to 5, 10, 20, 30, 40 and 50 ppm NO_2_ at room temperature.

Figure 10 shows the dynamic response curves of sensors in response to 5–50 ppm NO_2_. It can be seen that the responses of the sensors increased significantly with the increase of NO_2_ concentration. There was baseline drift at the place with high concentration. Compared with pure PEDOT, CoCe-BTC/PEDOT had a higher response to NO_2_ in the range of 10–50 ppm.

**Figure 11:**
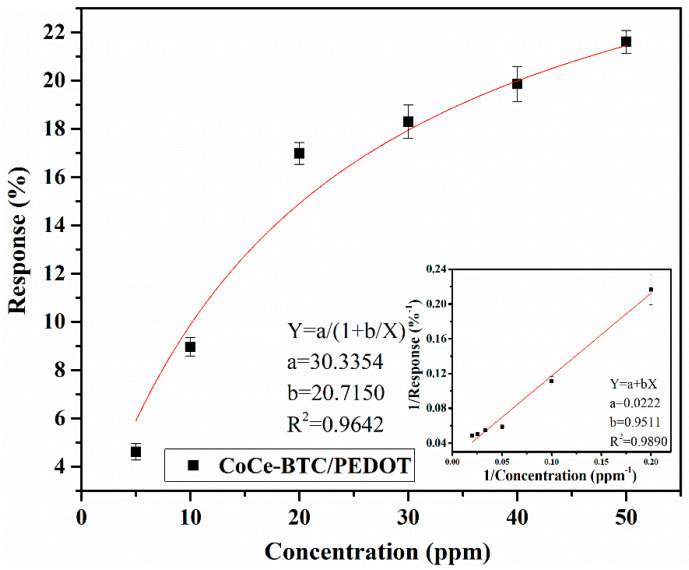
Response of the CoCe-BTC/PEDOT gas sensors to 5–50 ppm NO_2_ at room temperature (inset: linear fitting curve of R^−1^ vs. C^−1^, n = 3).

A Langmuir adsorption isotherm, R = a/(1 + b/C), was used to fit with the concentration (C)–response (R) data (R^2^ = 0.9642). The linear fitting of R^−1^–C^−1^ (R^2^ = 0.9890) indicated that the Langmuir adsorption isotherm could well describe the adsorption of NO_2_ on the sensor (Figure 11). The adsorption sites were not completely covered, and saturation of the response gradually occurred at high concentrations due to the lack of adsorption sites. The comparison of the gas sensing performance of the sensor in this work with other NO_2_ sensors previously reported is summarized in Table 1 [16,17,18,19].

### 3.4. Selectivity of Sensors

**Figure 12:**
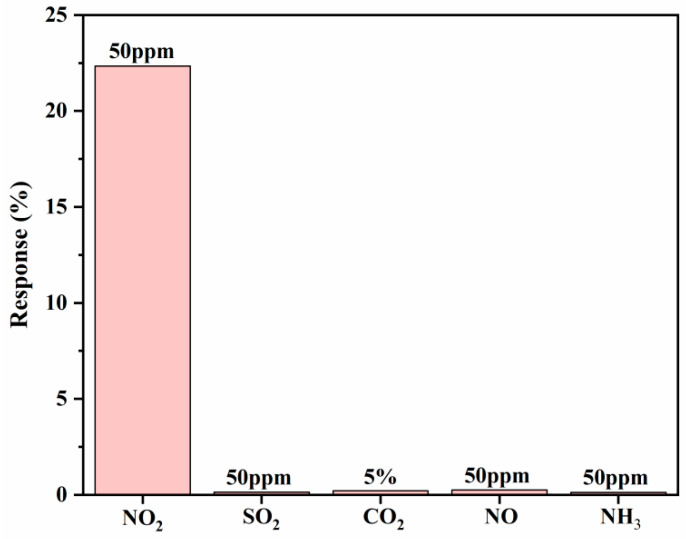
Selectivity histogram of CoCe-BTC/PEDOT film.

SO_2_, CO_2_, NO and NH_3_ were selected here to test the selectivity of sensors. The response of CoCe-BTC/PEDOT to SO_2_, CO_2_, NO and NH_3_ was less than 0.5% (Figure 12), which means that CoCe-BTC/PEDOT had great selectivity for NO_2_.

CoCe-BTC exhibited p-type semiconductor behavior, and the adsorption and desorption of gas molecules on the surface changed its resistance. It had high density and evenly distributed active metal sites. When contacting the oxidative gas NO_2_, there was a conversion of Ce^3+^/Ce^4+^ and Co^2+^/Co^3+^. CoCe-BTC released electrons, creating holes in its valence band, which reduced its resistance. The greater the concentration of NO_2_, the more electrons transferred, and the greater the reduction of resistance. In addition, the synergistic action of Co and Ce (Ce^4+^+Co^3+^→Ce^3+^+Co^2+^) greatly improved its charge transfer rate [20]. Bimetallic MOFs had better conductivity compared with single metal MOFs, which is conducive to reducing the surface activation energy of MOFs and enhancing the surface reaction between materials and analytes.

PEDOT is a p-type semiconductor with reduced resistance when exposed to oxidative gas NO_2_. The response trends of CoCe-BTC and PEDOT were the same. After recombination, electrons could be transferred from PEDOT to Ce^4+^ and Co^3+^, resulting in the formation of Ce^3+^ and Co^2+^. Then electrons were transferred from Ce^3+^ and Co^2+^ to the adsorbed NO_2_, which promoted the release of electrons and decreased the resistance. The two materials exert synergy effect, resulting in a significant improvement in gas sensitivity response.

## 4. Conclusions

In summary, a columnar bimetal MOF—CoCe-BTC was synthesized by the water bath method, and a CoCe-BTC/PEDOT composite film sensor was prepared by the spin coating method, which improved the conductivity of CoCe-BTC and realized the room temperature detection of NO_2_. The response of the CoCe-BTC/PEDOT composite film sensor to NO_2_ gas is 1.2 times higher than that of pure PEDOT. The relationship between the response and NO_2_ concentration (5 ppm–50 ppm) conforms to the Langmuir adsorption isotherm and has good repeatability and selectivity (SO_2_, CO_2_, NO, NH_3_ as interference gas). The modification of bimetal MOFs with conductive polymer not only improves the conductivity of the material but also can be used as sensitive material for chemical resistance gas sensors.

## Figures and Tables

**Table 1:** Comparison of the sensing performance of NO_2_ sensor.

Materials	Gas Concentration (ppm)	Response	Response Time (s)	Recovery Time (s)	References
ZnO nanorods	1	1.6 ^a^	-	-	[16]
In_2_O_3_/rGO	30	8.25 ^a^	240	1440	[17]
MoS_2_-Au	2.5	0.30 ^b^	240	840	[18]
In_2_O_3_/MoS_2_	30	0.17 ^b^	-	-	[19]
PEDOT	50	0.18 ^b^	322	896	This work
CeCo-BTC/PEDOT	50	0.22 ^b^	299	847	This work

^a^ S = $R_{a}{/R}_{g}$, ^b^ S = ${(R}_{a}-R_{g}{)/R}_{a}.$

## Data Availability

The data presented in this study are available on request from the corresponding author.
